# Dose escalation of a curcuminoid formulation

**DOI:** 10.1186/1472-6882-6-10

**Published:** 2006-03-17

**Authors:** Christopher D Lao, Mack T Ruffin, Daniel Normolle, Dennis D Heath, Sandra I Murray, Joanne M Bailey, Martha E Boggs, James Crowell, Cheryl L Rock, Dean E Brenner

**Affiliations:** 1 Division of Hematology-Oncology, Department of Internal Medicine, University of Michigan,2150 CCGC, Ann Arbor, MI 48109-0930, USA; 2 Department of Family Medicine, University of Michigan, 1018 Fuller St., Ann Arbor, MI 48109-0708; 3 Biostatistics Core, Cancer Center, University of Michigan, Room 8D22, 300 North Ingalls Bldg., Ann Arbor, MI 48109-0473, USA; 4 Cancer Prevention and Control Program. University of California, San Diego,9500 Gilman Drive, Dept. 0901, La Jolla, CA 92093-0901, USA; 5 Division of Cancer Prevention, National Cancer Institute, National Institute of Health,9000 Rockville Pike, Bethesda, MD 20892, USA; 6 Department of Family and Preventive Medicine, University of California, San Diego,9500 Gilman Drive, Dept. 0901, La Jolla, CA 92093-0901, USA

## Abstract

**Background:**

Curcumin is the major yellow pigment extracted from turmeric, a commonly-used spice in India and Southeast Asia that has broad anticarcinogenic and cancer chemopreventive potential. However, few systematic studies of curcumin's pharmacology and toxicology in humans have been performed.

**Methods:**

A dose escalation study was conducted to determine the maximum tolerated dose and safety of a single dose of standardized powder extract, uniformly milled curcumin (*C*^3 ^*Complex*™, Sabinsa Corporation). Healthy volunteers were administered escalating doses from 500 to 12,000 mg.

**Results:**

Seven of twenty-four subjects (30%) experienced only minimal toxicity that did not appear to be dose-related. No curcumin was detected in the serum of subjects administered 500, 1,000, 2,000, 4,000, 6,000 or 8,000 mg. Low levels of curcumin were detected in two subjects administered 10,000 or 12,000 mg.

**Conclusion:**

The tolerance of curcumin in high single oral doses appears to be excellent. Given that achieving systemic bioavailability of curcumin or its metabolites may not be essential for colorectal cancer chemoprevention, these findings warrant further investigation for its utility as a long-term chemopreventive agent.

## Background

Curcumin is the major yellow pigment extracted from turmeric, a commonly-used spice derived from the rhizome of the herb *Curcuma longa*. In India and Southeast Asia, it has long been used as a treatment for inflammation, skin wounds and tumors [1,2]. The clinical efficacy of curcumin has yet to be confirmed, but its cancer chemopreventive potential is demonstrated by the anticarcinogenic effects in cell culture and animal models of skin, breast, and gastrointestinal carcinogenesis [3-6].

Curcumin appears to be safe in both large and small animal models, but few systematic studies of curcumin's pharmacology and toxicology in humans have been performed [7-9]. Minimal toxicity of doses up to 8,000 mg have been described in humans [7,8] and to date, no maximum tolerated dose has been defined. Despite curcumin's apparent poor bioavailability [7,8], peak plasma concentration has been identified 1 to 2 hours after single dose oral administration of 4,000 mg and higher [7]. Based on this information, we performed a dose escalation study to determine the maximum tolerated dose, safety profile, and resultant serum concentration of a single dose of standardized powder extract obtained from Alleppey finger turmeric (*C*^3 ^*Complex*™, Sabinsa Corporation).

## Methods

### Study population and design

Eligible participants were male and female volunteers, 18 years of age or older with normal organ function who had not consumed any curcumin-rich foods to their knowledge within the previous 14 days. Subjects completed a food checklist to verify that they were not consuming curcumin-rich foods. After written informed consent was obtained, three subjects were entered consecutively at dose levels from 500 to 12,000 mg. Subjects took the dose with 8 fl. oz. of water followed by a standard meal containing dietary fat (providing 34 g or 42 g fat, per 2200 kcal/day or 2500 kcal/day meal plan, respectively).

Safety was assessed for 72 hours following the curcumin dose. Toxicities were graded based on National Cancer Institute, Common Toxicity Criteria version 2.0 [10]. The maximum tolerated dose was the highest dose which did not cause escalation to cease.

The study protocol and the comprehensive written informed consent used in this study protocol were reviewed and approved by the University of Michigan Human Subject Review Board prior to the start of the study. This review panel is certified under the Federal Wide Assurance Of Protection For Human Subjects (FWA 00004969) that expires June 12, 2006. The investigators followed with annual reports, renewals, and approval throughout the duration of the study including the analysis of the data.

### Study drug

Curcumin was provided in a capsule form as a standardized powder extract obtained from Alleppey finger turmeric (*C*^3 ^*Complex*™, Sabinsa Corporation). It contains a minimum 95% concentration of three curcuminoids: curcumin, bisdemethoxycurcumin, and demethoxycurcumin.

### Serum curcumin analysis

Blood specimens from all subjects on the escalation phase were obtained just prior to dosing, and 1, 2 and 4 hours after completing dosing at each dose level tested. The samples were assayed using our published methods [11]. Briefly, a total of 400 μL plasma were extracted with 500 μL of ethyl acetate/methanol reagent. The recovered solution was dried under a stream of nitrogen gas, resuspended in 200 μL of mobile phase reagent, and assayed using high performance liquid chromatography (HLPC). HPLC conditions were: isocratic mobile phase of acetonitrile/methanol/water/acetic acid (40:23:36:1, v/v/v/v), at a flow rate of 1.0 ml/min; column: Waters SymmetryShield, 3.9 × 150 mm, 5 μm; UV detection at 262 nm. Curcumin detection was found to be linear within a range of 0.2–7.0 μg/mL, with a limit of detection of 0.031 μg/mL. Between run coefficient of variation (CV) was <7.5.

## Results

Thirty-four participants signed informed consents. Twenty-four completed the trial and were eligible for this analysis. Of the twenty-four participants analyzed in this trial, thirteen men and eleven women with mean age of 34 years (range 19–74 years) were enrolled in the study. The racial distribution was 18 Caucasians and 6 African-Americans. Seven adverse events occurred and all were grade 1 as summarized in Table 1. No toxicity appeared to be dose related.

**Table 1:** All adverse events by dose levels

**Dose level^a^**	**Type**	**No. of events**	**Toxicity grade^b^**
1000 mg	Diarrhea	1	1
4000 mg	Headache	1	1
8000 mg	Rash	1	1
	Yellow Stool	1	1
10000 mg	Yellow Stool	1	1
	Headache	1	1
12000 mg	Diarrhea	1	1

^a ^Total of 3 subjects at each dose level

^b ^National Cancer Institute, Common Toxicity Criteria v.2.0 [10]

No curcumin was detected in serum of participants administered 500, 1,000, 2,000, 4,000, 6,000 or 8,000 mg. Table 2 shows the concentrations of curcumin in two subjects (1 taking 10,000 mg, and 1 taking 12,000 mg). No plasma concentrations of curcumin were detected in the remaining subjects at the 10,000 or 12,000 mg dose levels.

**Table 2:** Serum curcumin levels in ng/ml for two subjects

**Dose**	**Baseline**	**One hour**	**Two hour**	**Four hour**
10000 mg	approx. 6.0	30.4	39.5	50.5
12000 mg	approx. trace	29.7	57.6	51.2

## Discussion

Curcumin has broad spectrum chemopreventive activity in preclinical studies and appears to be safe in animal and human studies [3,12,13]. For chemopreventive interventions to be successful, they must be provided in doses that are effective, but be nearly free of toxicities. Several studies have demonstrated minimal toxicity with moderate doses of curcumin given in various formulations. Soni and Kuttan [14] administered 500 mg capsules of a 98% pure curcumin formulation to 10 volunteers daily for 7 days and reported no clinical toxicity. Two trials evaluating the efficacy of turmeric or curcumin for the treatment of arthritis or postoperative inflammation found that doses of 1,200 to 2,100 mg of curcumin per day for 2–6 weeks were without adverse effects [15,16]. Cheng et al [7] reported no treatment-related toxicity up to 8,000 mg/day using a 99.3% pure curcumin tablet (500 mg of curcumin in each tablet). However, doses up to 12,000 mg were unacceptable to patients due to the bulky volume of the tablets. In the present study, we recognized minimal toxicity up to 12,000 mg in a single dose of standardized powder extract (*C*^3 ^*Complex*™, Sabinsa Corporation) obtained from Alleppey finger turmeric (Table 1). Thus, to our knowledge, no maximum tolerated dose has been identified in humans, although successful administration may be affected by differences in drug formulation.

Low serum levels of curcumin after single dose administration is consistent with the previously reported pharmacokinetic studies in animals and humans [7-9,17-20]. Wahlstrom et al [18] administered 1 g/kg to Sprague-Dawley rats and found 65–85% of the dose excreted unchanged in the feces with negligible amounts in the urine. Studies with deuterium and tritium-labeled curcumin given orally to rats resulted in most of the detectable radioactivity in the feces [18,20]. Negligible amounts of radioactivity were detectable in plasma when smaller doses of [^3^H]-curcumin were administered [20]. Sharma et al [8] administered *Curcuma *extract 440 to 2,200 mg/day (36 to 180 mg of curcumin) for up to 29 days to patients with advanced colorectal cancer and failed to detect curcumin or its metabolites in blood or urine. Serum concentrations may be dependent on the dose that is administered. Cheng et al [7] determined the concentration of curcumin by HPLC in 25 subjects with high risk cancer lesions. The peak serum concentration after 4,000, 6,000, and 8,000 mg were 0.51 ± 0.11 μM, 0.64 ± 0.06 μM, and 1.77 ± 1.87 μM, respectively, but doses below 4,000 mg were barely detectable. These findings are consistent with the present study, in which low levels of curcumin were measured only at doses ≥8,000 mg (Table 2).

Low serum levels of curcumin may be due in part to the extensive intestinal and hepatic metabolic biotransformation. Preclinical work has demonstrated that avid sulfation, glucuronidation, and reduction of curcumin occurs in the gastrointestinal tracts of rats and humans [19,21-23]. A challenge for future chemopreventive strategies lies in the conflicting evidence of the biological effects of the resultant metabolites. For example, lymphocytic glutathione *S*-transferase activity, a potential surrogate biomarker of curcumin activity, was significantly decreased in patients taking 440 mg/day despite the lack of measurable serum curcumin [8]. This finding suggests that curcuminoid metabolites, which may not have been detected, resulted in a systemic biological effect. In contrast, experiments by Ireson et al [24] suggest that the metabolism of curcumin generates species with reduced ability to inhibit COX-2 expression compared to the parent compound. Given these conflicting data, the poor bioavailability of curcumin, and the fact that the gastrointestinal tract is exposed to the greatest concentration of unmetabolized curcumin, colorectal cancer chemoprevention is the most attractive area for future efforts.

To further elucidate the cause of low serum curcumin levels after large dose consumption in the present study, the curcumin capsules were analyzed. Each capsule was found to be 75% curcumin, 23% demethoxycurcumin and 2% bisdemethoxycurcumin, which is consistent with other commercial curcuminoid products. However, the curcumin activity as measured by HPLC area count for a weighed portion of capsular content was 66% less than an equivalent weighed portion of a previously supplied, pure curcumin powder (unpublished data). Thus, curcumin bioavailability in humans and biologic activity might differ based upon the formulation used.

## Conclusion

The tolerance of curcumin (*C*^3 ^*Complex*™, Sabinsa Corporation) in single oral doses up to 12,000 mg appears to be excellent and warrants further investigation for its utility as a long-term chemopreventive intervention. Despite the lack of a defined maximum tolerated dose, the more important issue in chemopreventive investigation lies in determining the biologically effective dose. Future clinical investigation is required to determine such a dose. Poor bioavailability from the gut limits the systemic cancer preventive activity of curcumin and higher doses, as used in this study, may be required to overcome intestinal metabolism and achieve systemic effects. However, further investigation to determine the direct effects of curcumin on colonic mucosa are warranted to adequately assess the efficacy of curcumin as a colorectal cancer chemopreventive agent.

## Competing interests

The author(s) declare that they have no competing interests.

## Authors' contributions

CL, MR, DB participated in concept development; study design, implementation, and coordination; and helped to draft the manuscript. DN participated in the design of the study and performed the statistical analysis. DH developed and performed the HPLC assays. CR participated in the design of the study and also developed and performed the HPLC assays. SM, JB, MB participated in the design of the study and implementation of study protocol. JC participated in concept and study design development.

## Pre-publication history

The pre-publication history for this paper can be accessed here:


